# Frequency- and Area-Specific Phase Entrainment of Intrinsic Cortical Oscillations by Repetitive Transcranial Magnetic Stimulation

**DOI:** 10.3389/fnhum.2021.608947

**Published:** 2021-03-12

**Authors:** Yuka O. Okazaki, Yumi Nakagawa, Yuji Mizuno, Takashi Hanakawa, Keiichi Kitajo

**Affiliations:** ^1^RIKEN CBS-TOYOTA Collaboration Center, RIKEN Center for Brain Science, Wako, Japan; ^2^Division of Neural Dynamics, Department of System Neuroscience, National Institute for Physiological Sciences, National Institutes of Natural Sciences, Okazaki, Japan; ^3^Department of Physiological Sciences, School of Life Science, The Graduate University for Advanced Studies (SOKENDAI), Okazaki, Japan; ^4^Integrative Brain Imaging Center, National Center of Neurology and Psychiatry, Kodaira, Japan; ^5^Department of Integrated Neuroanatomy and Neuroimaging, Graduate School of Medicine, Kyoto University, Kyoto, Japan

**Keywords:** rTMS-EEG, phase entrainment, natural frequency, visual cortex, motor cortex, phase locking

## Abstract

Synchronous oscillations are ubiquitous throughout the cortex, but the frequency of oscillations differs from area to area. To elucidate the mechanistic architectures underlying various rhythmic activities, we tested whether spontaneous neural oscillations in different local cortical areas and large-scale networks can be phase-entrained by direct perturbation with distinct frequencies of repetitive transcranial magnetic stimulation (rTMS). While recording the electroencephalogram (EEG), we applied single-pulse TMS (sp-TMS) and rTMS at 5, 11, and 23 Hz over the motor or visual cortex. We assessed local and global modulation of phase dynamics using the phase-locking factor (PLF). sp-TMS to the motor and the visual cortex triggered a transient increase in PLF in distinct frequencies that peaked at 21 and 8 Hz, respectively. rTMS at 23 Hz over the motor cortex and 11 Hz over the visual cortex induced a prominent and progressive increase in PLF that lasted for a few cycles after the termination of rTMS. Moreover, the local increase in PLF propagated to other cortical areas. These results suggest that distinct cortical areas have area-specific oscillatory frequencies, and the manipulation of oscillations in local areas impacts other areas through the large-scale oscillatory network with the corresponding frequency specificity. We speculate that rTMS that is close to area-specific frequencies (natural frequencies) enables direct manipulation of brain dynamics and is thus useful for investigating the causal roles of synchronous neural oscillations. Moreover, this technique could be used to treat clinical symptoms associated with impaired oscillations and synchrony.

## Introduction

Large-scale phase synchronization of neural oscillations plays a role in linking task-relevant brain regions associated with information processing (Varela et al., 2001; Fries, 2005). Although phase synchronization of neural oscillations is ubiquitous in the brain, the frequency of synchronous oscillations varies across distinct brain regions and networks (Siegel et al., 2012), and is presumably associated with segregated and integrated networks that mediate various functions, such as face perception (Rodriguez et al., 1999), selective attention (Doesburg et al., 2008), and working memory (Kawasaki et al., 2010).

Frequency-specific network structures characterized by phase synchronization also exist in the resting state (Hillebrand et al., 2012; Hipp et al., 2012) and are spatially consistent with the task-driven networks, as demonstrated by fMRI studies (Smith et al., 2009; Deco and Corbetta, 2011). Moreover, research in animals has shown that sensory-evoked population firing patterns are geometrically confined to subregions of the neuronal state space delineated by spontaneous activity (Luczak et al., 2009). These studies suggest that the spatiotemporal patterns of spontaneous neural activity reflect a wide repertoire of neural dynamics, and may constrain the task-related neural dynamics. However, few mechanistic details are known about the various sets of rhythmic activity in different cortical areas.

Non-invasive brain stimulation techniques such as transcranial magnetic and electrical stimulation (TMS and TES) have emerged as promising manipulative tools, enabling direct perturbation of local brain areas. TMS, in particular, can be used to target spatially confined regions involved in generating oscillations. The use of TMS in combination with EEG allows us to study the mechanisms underlying changes in response measures (excitability and connectivity) to perturbations within brain networks. In a previous TMS-EEG study, we showed that the phase synchronization of the intercortical networks changes dynamically according to the attentional state (Okazaki et al., 2020). In addition, single-pulse TMS has been shown to directly interfere with the phase dynamics of oscillations and trigger a transient phase reset of intrinsic oscillations in visual areas (Kawasaki et al., 2014). It is noteworthy that when single-pulse TMS was applied to different cortical regions that constitute specific corticothalamic networks, oscillations induced in occipital, parietal, and frontal cortices fell into distinct frequency bands (Rosanova et al., 2009). This indicates that each corticothalamic network has its own characteristic intrinsic frequency, or “natural frequency.” Given the results of single pulse-induced brain oscillations, entrainment of the oscillatory phase can be predicted when further TMS pulses are applied in phase with the induced oscillations (Lakatos et al., 2019). Consequently, the amplitude of oscillations gradually increases as more and more intrinsic neural oscillators are entrained to the repetitive TMS pulses (Thut et al., 2011a). In line with this hypothesis, Thut et al. demonstrated local enhancement of alpha oscillations by applying alpha-frequency train of TMS pulses to the parietal cortex (Thut et al., 2011b).

To our knowledge, no previous study has applied rhythmic stimulation at multiple frequencies to distinct cortical regions. However, based on the above-mentioned evidence (Rosanova et al., 2009; Thut et al., 2011b), it seems probable that distinct cortical regions have their own optimal frequencies for the phase entrainment of intrinsic neural oscillations. We, therefore, hypothesized that there exist region-specific differences in the frequency characteristics of phase entrainment of intrinsic oscillations by external stimulation. Moreover, it is not clear how locally entrained oscillations impact oscillations in other cortical regions *via* large-scale functional networks. Given that local brain areas that are targeted by periodic stimulation are globally coupled to oscillatory modules in other brain regions, we also hypothesized that locally entrained oscillations propagate to other connected areas with specific frequency characteristics. To address these hypotheses, we measured phase dynamics using scalp electroencephalography (EEG) while applying rTMS to either the motor or visual cortex at theta- (5 Hz), alpha- (11 Hz), or beta- (23 Hz) band frequencies.

## Materials and Methods

### Participants

Fourteen healthy right-handed participants (two females and 12 males aged 30.8 ± 5.5 years, mean ± SD) provided informed, written consent to participate in the study. This study was conducted in accordance with the declaration of Helsinki, and was approved by the RIKEN Ethics Committee.

### TMS-EEG Experiments

**Figure 1:**
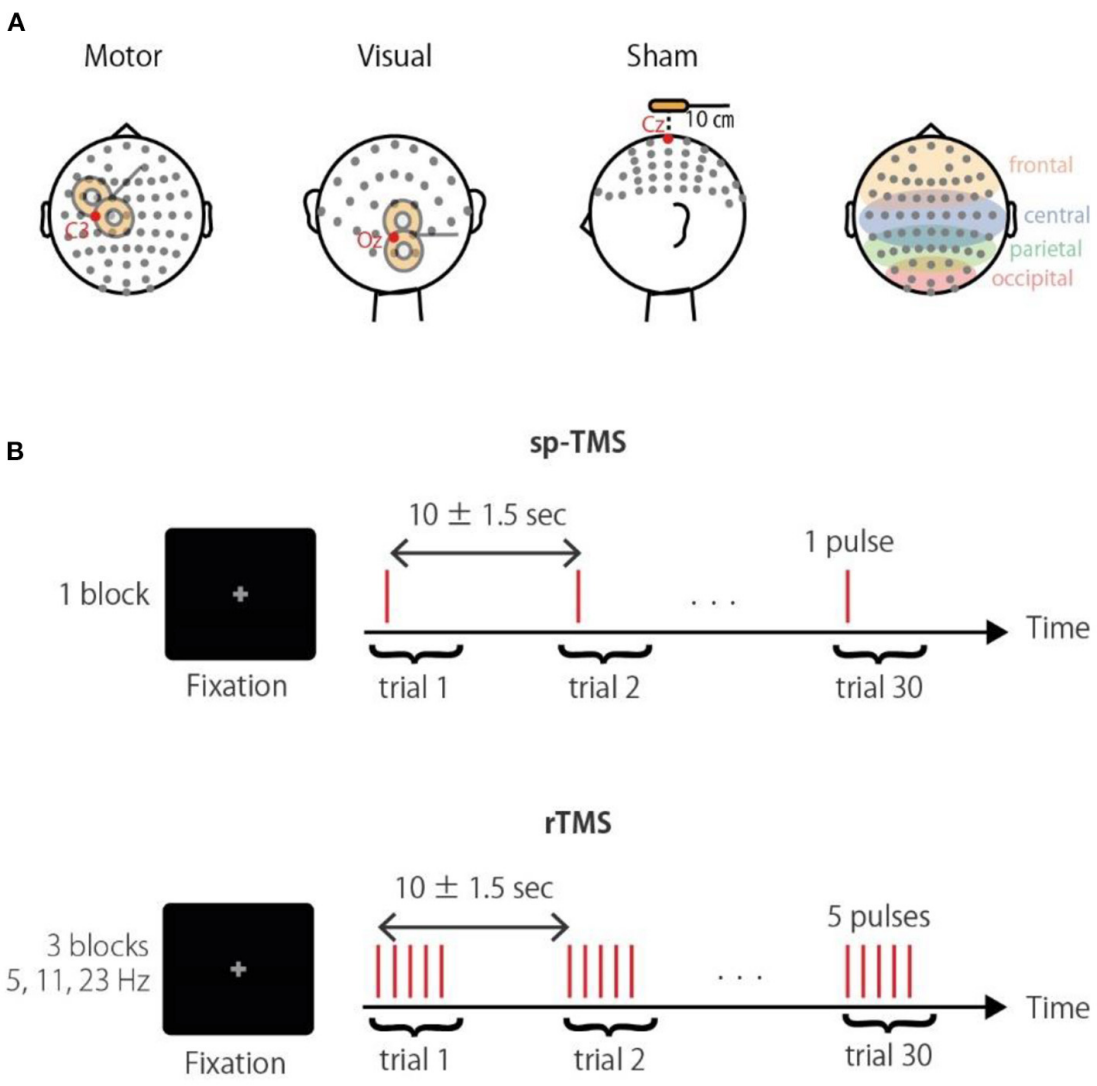
Experimental paradigm. **(A)** Schematic illustration of the stimulation sites. Corresponding electrodes to the occipital, parietal, central, and frontal areas are highlighted in the right panel. **(B)** The TMS conditions were a single pulse (sp-TMS), and five pulses at 5, 11, or 23 Hz (rTMS). The TMS conditions were provided in blocks with 30 repetitions. Participants had to fixate a central cross during trials.

TMS was applied in a biphasic pulse configuration by a Magstim Rapid unit with a figure-of-eight coil (Double 70 mm Alpha coil; Magstim, UK). Stimulation was applied over either the visual or motor regions, or a sham control location (Figure 1A). For the sham stimulation, the coil was positioned 10 cm above the vertex with the coil handle oriented in a posterior direction. Thus, sham stimulation produced a TMS “click” sound that occurred at the same frequency as that of the real TMS session, but without cortical stimulation; in all conditions, the click sound was attenuated by earplugs. For stimulation of visual regions, the coil was located centrally between the Oz and O2 electrodes with the coil handle pointing rightward. For stimulation of motor regions, the coil position was determined individually at the “hotspot” that activated the right first dorsal interosseous (FDI) (approximately at the C3 electrode, with the handle perpendicular to the central sulcus). Posterior-anterior current flow for the second half-wave of the biphasic pulse induces an effective current flow for axon depolarization (Kammer et al., 2001). In terms of noise reduction (Komssi et al., 2004; Litvak et al., 2007) and induction of recordable phase reset (Kawasaki et al., 2014), a stimulation intensity of 90% of the FDI active motor threshold was used in all conditions. To reduce the noise induced in the electrode lead wire, the lead wire and the TMS coil handle are arranged orthogonally.

### EEG Recordings

During stimulation, EEG (left earlobe reference; ground AFz was continuously recorded at a 5 kHz sampling rate (filtering: DC to 1,000 Hz) from 63 scalp sites *via* Ag/AgCl TMS-compatible electrodes mounted on a 10/10 system EasyCap (EASYCAP GmbH, Germany). Horizontal and vertical electrooculography (EOG; ground electrode, left mastoid) signals were simultaneously recorded. Electrode impedance was maintained below 10 kΩ. The EEG and EOG signals were amplified using a BrainAmp MR plus system (Brain Products GmbH, Germany).

### Experimental Procedure

Three TMS-EEG sessions with different stimulation sites (visual, motor, and sham) were conducted in a semi-random order that was counterbalanced across participants. Each session consisted of the four following blocks: a single pulse (sp-TMS), and a 5-pulse train at 5 Hz (θ-rTMS), 11 Hz (α-rTMS), and 23 Hz (β-rTMS). Thus, the duration from the first to the last pulse of rTMS was 800, ~364, and ~174 ms, respectively. These frequencies were chosen to differentiate the entrained frequencies, including their harmonics. The block order was also counterbalanced. Each block comprised 30 trains with an inter-train interval of 10 ± 1.5 s. Participants were fixated on a centrally presented gray cross on a black background during each block (Figure 1B). Stimulus delivery was controlled using Psychtoolbox-3 (Brainard, 1997; Pelli, 1997; Kleiner et al., 2007).

### Preprocessing

EEG data were analyzed using MATLAB (Mathworks, USA) scripts that were developed in-house with FieldTrip (Oostenveld et al., 2011) and EEGLAB (Delorme and Makeig, 2004). For segmented (from 2 s before the first pulse to 3 s after the last pulse) and re-referenced (the average of the left and right earlobe electrode signals) EEG data, TMS artifacts and noisy epochs were removed by performing the following steps according to Herring et al. (2015) (see also http://www.fieldtriptoolbox.org/tutorial/tms-eeg for the detailed procedure). First, we linearly interpolated the 40 samples (8 ms) after TMS onset, which is the period that usually shows excessive TMS artifacts. In the case of artifacts occurring after 8 ms, we used interpolation of 60 samples (12 ms). Second, we attenuated the exponentially decaying TMS artifacts using independent component analysis (ICA) (Korhonen et al., 2011). Independent components with extremely large amplitudes, i.e., those with maximum *z*-score values >1.65 between 0 and 100 ms, were removed. Third, we discarded epochs within 1 s pre- or post-stimulation in which the EEG amplitude exceeded 200 μV; on average, 24.8 ± 2.5 [SD] trials remained in each condition. Then, we applied current source density (CSD) transformation to the EEG voltage map using the spherical-spline surface Laplacian algorithm to attenuate the effects of volume conduction using the CSD Toolbox (Perrin et al., 1989; Kayser and Tenke, 2006). Finally, the data were downsampled to 1,000 Hz. The artifact-corrected TMS-EEG data were also analyzed in our previous study, which had a different purpose (i.e., probing phase-amplitude coupling) (Glim et al., 2019). Data and code are available upon reasonable request to the corresponding author.

### Time-Frequency Analysis

The time–frequency representations (TFRs) of the instantaneous amplitude and phase were obtained using a wavelet transform at a center frequency *f* and time *t*, with standard deviations σ_*f*_ = *4f/m* and σ_*t*_ = *m/2*π*f* (Lachaux et al., 2000). The constant *m* was set to 3. In general, the phase entrainment of oscillations is achieved through the gradual phase alignment of the oscillations by periodic external inputs. We expected that the phase of EEG oscillations would be gradually aligned *via* successive pulses of rTMS, and the degree of phase alignment would be assessed as the phase consistency of EEG signals across trials. We also expected that the phase-aligned oscillations would persist for a short time even after rTMS was terminated (Thut et al., 2011a). Here, the phase entrainment was defined as the phase-locking factor (PLF) (Tallon-Baudry et al., 1996), which evaluates phase consistency across trials. If the phase shifts toward a particular phase due to the TMS pulses, the phase will be consistent across trials and the PLF will increase.

$$PLF_{m}=\left|\frac{1}{N}\sum_{n=1}^{N}\exp(i(\varphi_{m,\;n\;}\left(f,t\right))\;\right|,$$

where φ_*m,n*_ is the instantaneous phase of the *n*th trial at electrode *m* and *N* denotes the total number of trials. Based on the circular statistics, Rayleigh's *Z* transform is used to test for circular uniformity, which takes into account the critical value bias according to the sample size. We applied it to PLF to compare between conditions with different numbers of trials due to the artifact removal procedure:

$$\begin{array}{l} ZPLF=N\times PLF^{2} \end{array}$$

(Fisher, 1993; Mazaheri and Jensen, 2006; Bonnefond and Jensen, 2012). The biased PLF and unbiased ZPLF according to the number of trials, which was limited due to the total duration of the experiment in the current study, were confirmed using our empirical EEG data (Supplementary Figure 1).

### Statistical Analysis

Significant differences in the ZPLF between TMS (sp-TMS and rTMS) and sham-TMS were determined by performing cluster-based permutation tests (Maris and Oostenveld, 2007), which evaluate comparisons between cluster-level statistics of the observed data matrices and those of the null distribution. First, ZPLF matrices (i.e., 63 electrodes, 3–45 Hz, −0.5 to 1.5 s) for the TMS and sham-TMS conditions were compared using a two-tailed paired *t*-test with a threshold uncorrected *p*-value < 0.05 to locate contiguous negative and positive clusters in the matrices. Cluster-level statistics were determined as the sum of the *t*-values within the cluster. Second, to generate a null distribution for the cluster-level statistics, the highest cluster-level statistic was identified from matrices in which two condition labels were randomly permuted within participants and iterated 500 times. Finally, a significant cluster level was defined in the observed data as the 97.5th percentile of the null distribution.

Next, we tested whether modulation of the ZPLF was more globally distributed at frequencies matching the stimulation frequency than at other frequencies. The number of significant electrodes, identified by the above cluster-permutation test, at each frequency and time was counted. Then, the numbers of significant electrodes at the stimulation frequency and at other frequencies were compared using the binomial test. All statistical results are summarized in Table 1.

**Table 1:** A summary of statistics for each figure.

**Figure #**	**Statistics**	**Comparison**	**Statistical representation**
2	Cluster-based permutation test	Real vs. Sham TMS	The sum of *t*-values within significant clusters
3	Dunnett's test	Baseline vs. Each pulse	The mean of ZPLFnorm
4	Cluster-based permutation test	Real vs. Sham TMS	The sum of *t*-values within significant clusters
5 A, C 5 B, D	Cluster-based permutation test Binomial test	Real vs. Sham TMS Real vs. Real TMS	The number of electrodes in significant clusters
6	Cluster-based permutation test	Real vs. Real TMS	The sum of *t*-values within significant clusters

## Results

### Phase Entrainment of Intrinsic Local Oscillations by Periodic Stimulation

**Figure 2:**
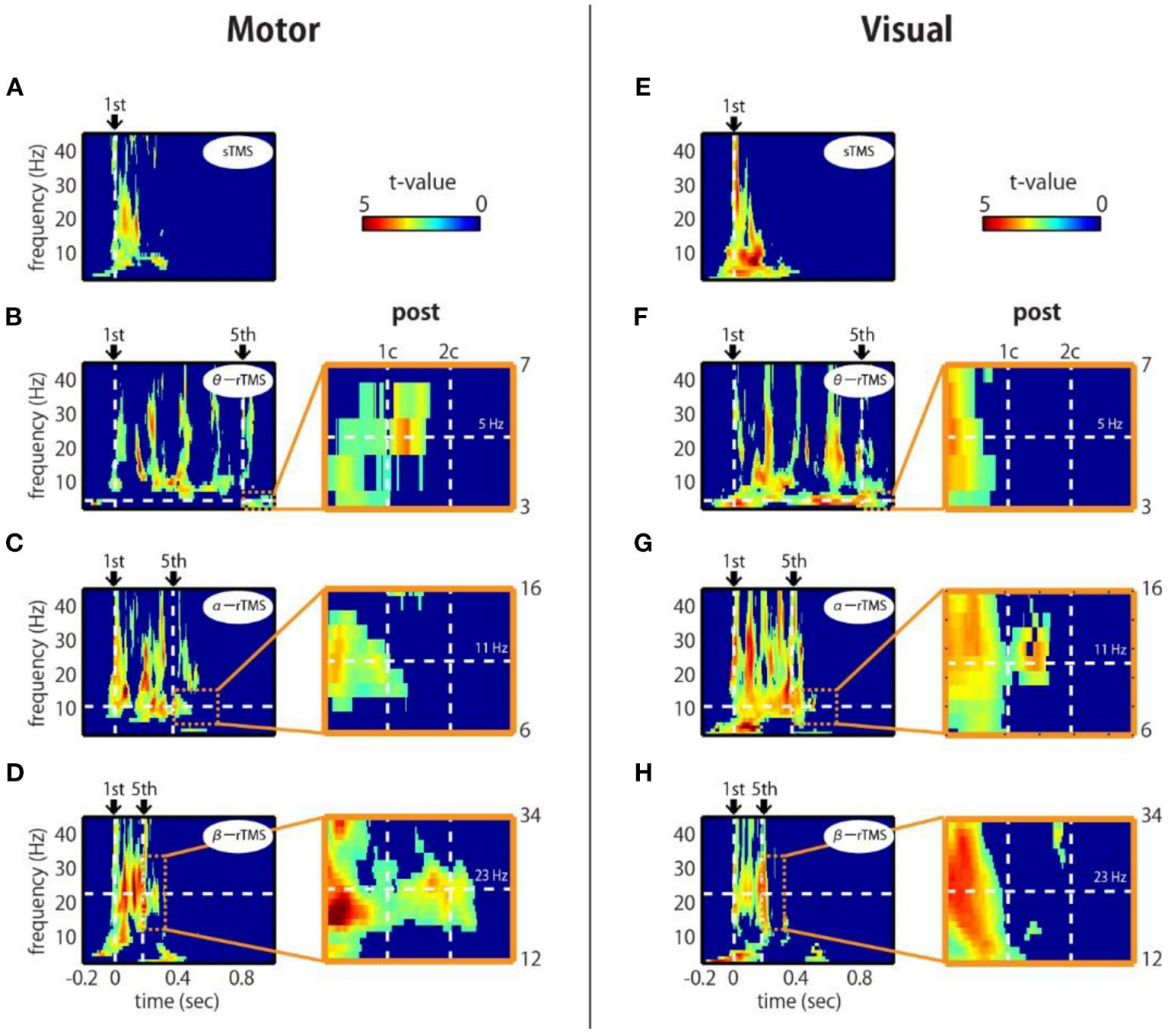
Time-frequency representations of ZPLF at the stimulated areas. Each map with *t-*values indicates significantly larger ZPLF for motor (C3) stimulation **(A–D)** and for visual (Oz) stimulation **(E–H)** than under the sham condition. The vertical lines in the left panels correspond to the first and last stimulation pulses. The horizontal lines indicate the stimulation frequency. Right panels are magnified views of three cycles after the end of rTMS. Vertical lines indicate the post-one cycle (1c) and post-two cycles (2c) for each stimulation frequency.

To assess the phase entrainment of neural oscillations by TMS, we first examined the TFR of the ZPLF for each stimulated area. The cluster-based permutation test revealed significant increases in phase locking by sp-TMS and rTMS (θ-rTMS, α-rTMS, β-rTMS) compared with the corresponding sham condition (*p* < 0.05, cluster-based permutation test). Figure 2 shows the significant *t*-values derived from cluster statistics (non-significant values are masked by zero). For stimulation over motor areas, sp-TMS induced a transient increase in the ZPLF at a broad frequency band, peaking at 21 Hz (Figure 2A). rTMS induced a prolonged increase in the ZPLF at the stimulation frequency (horizontal dashed line) during α-rTMS (Figure 2C) and β-rTMS (Figure 2D), but this effect was non-significant for the theta-band ZPLF during θ-rTMS (Figure 2B). For stimulation over visual areas, the peak response frequency to sp-TMS was 8 Hz (Figure 2E). In all rTMS conditions, a continuous increase in phase entrainment around the stimulation frequency was observed (Figures 2F–H).

Next, we examined whether the effects of phase locking across trials lasted, even after the rTMS train had terminated. If intrinsic oscillations are generated by self-sustaining systems without external input (Komssi et al., 2004), the phase locking should persist for a short time after the end of the stimulation train (Klimesch et al., 2004). We observed that the increase in the ZPLF lasted for more than two cycles after the last pulse of β-rTMS to the motor cortex (Figure 2D, magnified view) and 1.5 cycles after the last pulse of α-rTMS to the visual cortex (Figure 2G, magnified view). This lasting effect was not due to a signal processing limitation, i.e., spectral leakage in the time domain of the wavelet convolution, because its effect is theoretically <5% after one cycle and must be observed under all conditions. We also noted that these sustained frequencies (i.e., 23 Hz for the motor cortex and 11 Hz for the visual cortex) were slightly different from the frequencies of oscillations evoked by sp-TMS (i.e., peaks at 21 Hz for the motor cortex and 8 Hz for the visual cortex). Furthermore, we investigated whether the neural oscillations of participants with individual alpha frequencies (IAF) close to the α-rTMS frequency, i.e., 11 Hz, were more entrained. Participants who showed a prominent power peak were separated into low-IAF and high-IAF groups, as shown in Supplementary Figure 2A (four participants were excluded because they did not show a prominent power peak). Standardized by the mean and SD of the ZPLF over time points in the entire epoch interval, the ZPLFs were averaged for each group for α-rTMS over the visual cortex (Supplementary Figure 2B). Compared with the low-IAF group and the high-IAF group, the group whose intrinsic alpha frequency was closer to the stimulation frequency showed a longer phase entrainment effect. These results suggest that α-rTMS, which has a frequency sufficiently close (but not necessarily the same) to that of the natural frequency, was most effective for entraining intrinsic brain oscillations.

### Gradual Modulation of Phase-Locked Frequency Over TMS Pulses

**Figure 3:**
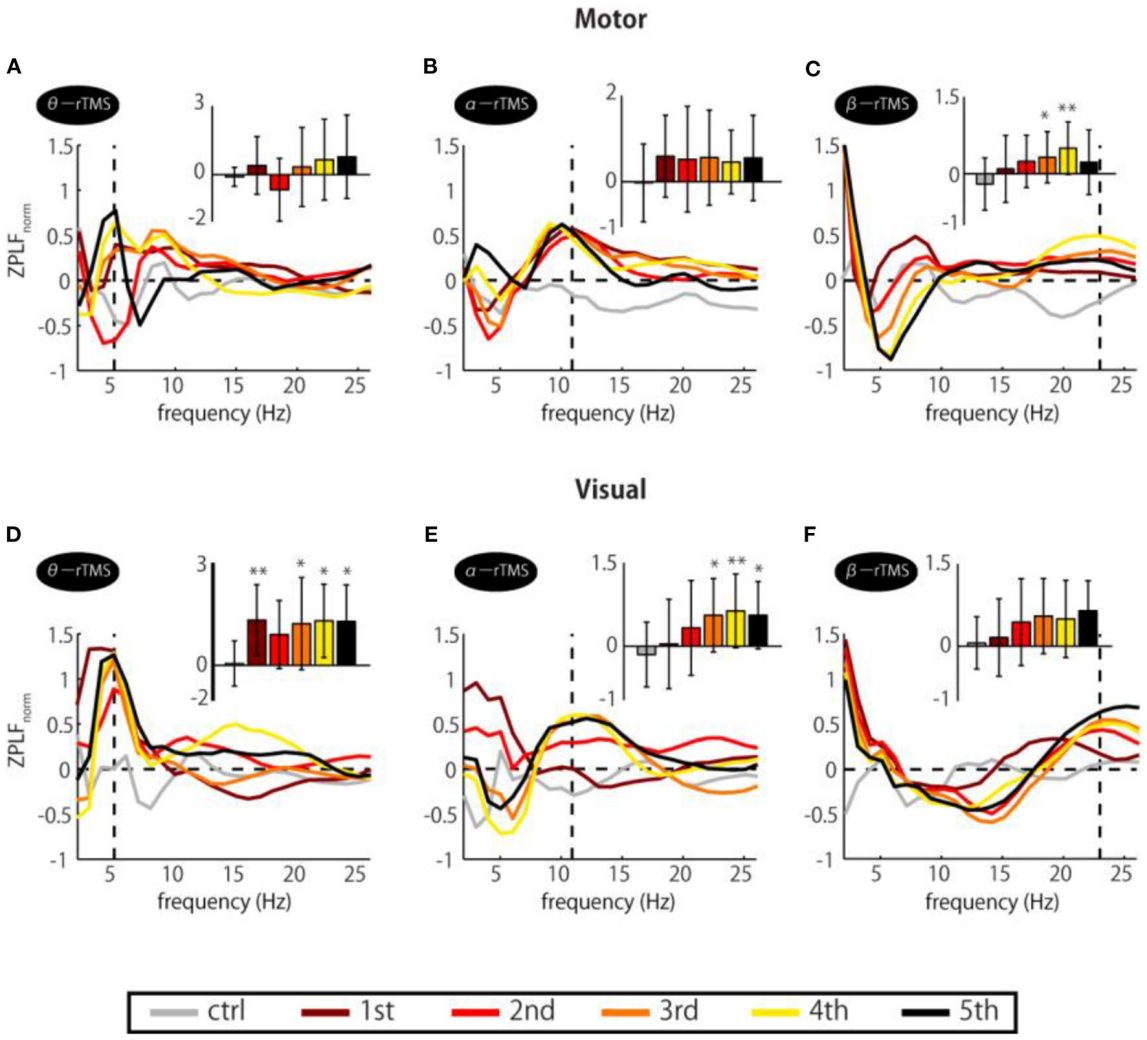
Changes in ZPLF_norm_ over TMS train. Each colored line corresponds to ZPLF_norm_ for each TMS pulse to the motor **(A–C)** and the visual cortex **(D–F)**. Together, they indicate which frequency was the most prominent during the train and how it varied with pulse repetition. The charts inset in each panel indicate the ZPLF_norm_ at the frequency corresponding to the rTMS stimulation frequency, i.e., the values of ZPLF_norm_ at the vertical dashed line. Significant changes from the baseline control period (ctrl) were assessed by Dunnett's test (*p < 0.05, **p < 0.01). The baseline was the mean value during the period from −5 to −2 stimulation-frequency cycles. Error bars: SD.

If entrainment is achieved through a phase alignment of ongoing oscillations to periodic force, successive phase alignment should result in gradual increases in the ZPLF (Thut et al., 2011a). As shown in Figure 2, increases in the ZPLF occurred not only around the stimulation frequency but also at a broad range of frequencies. Intriguingly, however, the most prominent phase-locked frequency varied over the course of the stimulation train. To measure which frequency showed the most prominent increase at each TMS pulse, we standardized the ZPLF using the mean and SD of the ZPLF over all frequencies (ZPLF_norm_). Figure 3 shows the ZPLF_norm_ averaged over ±0.5 cycles of each frequency for each TMS pulse. For example, for α-rTMS over the visual cortex, the dominant frequency was 5 Hz at the first pulse (brown line), but 11 Hz for the last three pulses (Figure 3E). The charts inset in each panel quantify, for each pulse in the train, whether the ZPLF_norm_ at the stimulation frequency was significantly larger than the ZPLF at baseline control (ctrl). Here, the ZPLF_norm_ at ctrl indicates the mean value of ZPLF_norm_ during the baseline period from −5 to −2 stimulation-frequency cycles. We applied a one-way ANOVA with the factor pulse (ctrl, 1st, 2nd, 3rd, 4th, 5th) to assess the effect of the pulse on phase locking. The one-way ANOVA revealed a significant main effect of the pulse on the ZPLF_norm_ in β-rTMS over the motor cortex [*F*_(5, 78)_ = 3.40, *p* = 0.008], but not in stimulation over the visual cortex [*F*_(5, 78)_ = 1.65, *p* = 0.156]. On the other hand, stimulation over the visual cortex showed a significant main effect of the pulse on the ZPLF_norm_ in α-rTMS [*F*_(5, 78)_ = 2.81, *p* = 0.022] and θ-rTMS [*F*_(5, 78)_ = 3.11, *p* = 0.013]. The Dunnett's *post-hoc* tests comparing each pulse with ctrl confirmed a significantly stronger (*p* < 0.05) phase locking than ctrl after the 3rd pulse of β-rTMS over the motor cortex (Figure 3C) and α-rTMS over the visual cortex (Figure 3E), although 5th pulse did not induce a maximum ZPLF_norm_. In addition, we confirmed the linearly increasing trend of the ZPLF as a function of the number of pulses using Pearson's correlation analysis (β-rTMS: *r* = 0.30, *p* = 0.005; α-rTMS: *r* = 0.39, *p* = 0.0002). Although there was a significant difference between pulses and ctrl in θ-rTMS over the visual cortex (Figure 3D), the phase locking to the stimulation frequency was more dominant than other frequencies for every TMS pulse in the train, i.e., it is not gradually increasing.

### Global Propagation of Phase Entrainment

**Figure 4:**
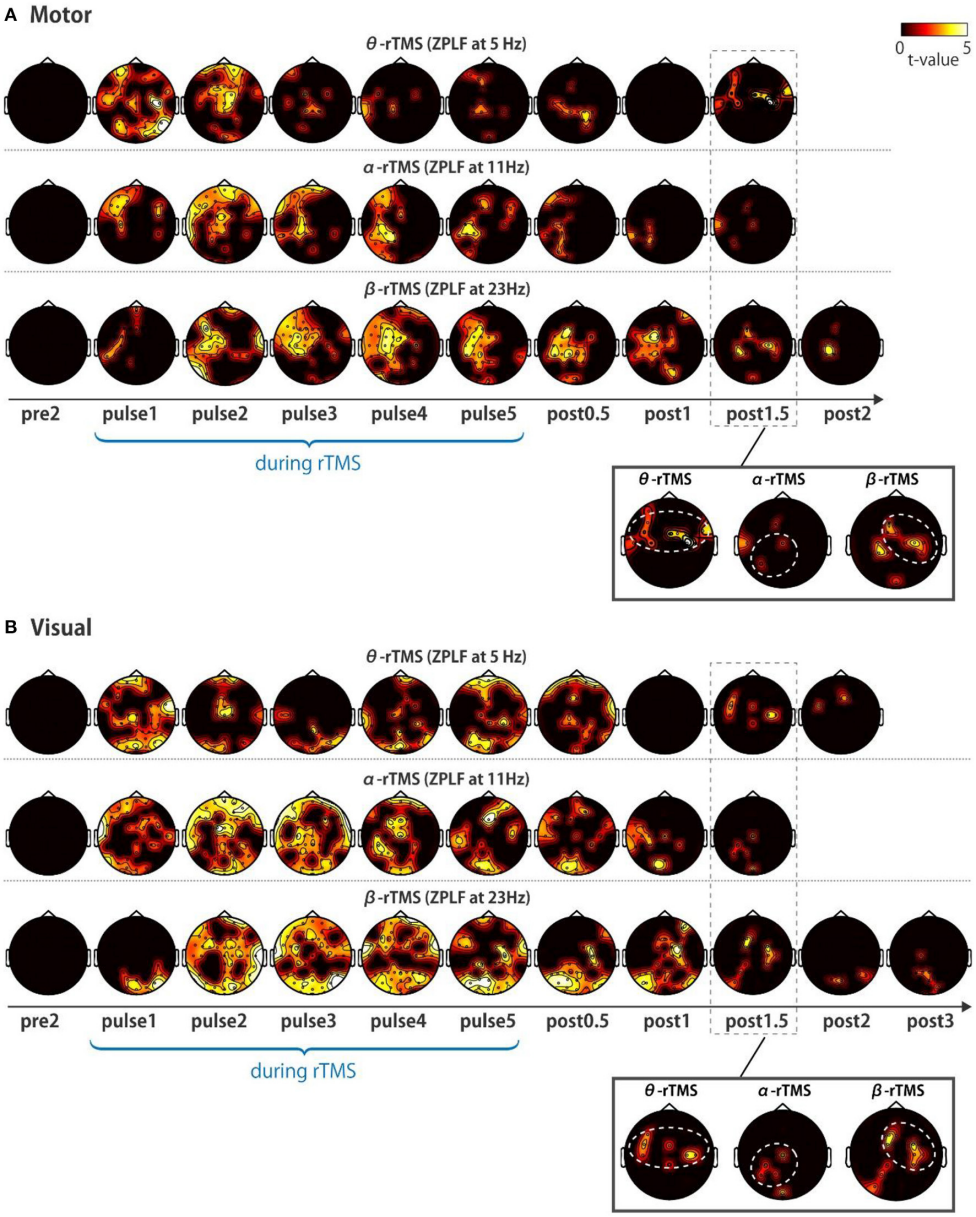
Topographic representation of ZPLF. Each map with *t*-values from two cycles pre-stimulation (pre2) to post-stimulation (post2) indicates significantly larger ZPLF for the motor **(A)**, and for visual stimulation **(B)** than under the sham condition (*p* < 0.05). The phase-locking of theta frequency oscillations in the frontoparietal, alpha frequency in the left occipitoparietal and beta frequency in the right motor areas are highlighted with white dotted circles in the magnified view of post 1.5 cycles.

Next, we addressed the question of whether local phase locking to rTMS propagates to other cortical areas. We first examined the spatial extent of the significant increases in the ZPLF by performing cluster-based permutation tests between TMS and sham conditions. Increases in the ZPLF with rTMS to the motor cortex were distributed to the ipsilateral hemisphere and partially extended to the contralateral hemisphere (Figure 4A). For stimulation over the visual cortex, rTMS resulted in widespread increases in the ZPLF reaching frontal areas (Figure 4B). The extensive phase locking during stimulation of the motor and visual cortices appears to be more localized for later pulses. Moreover, in several areas, phase locking persisted even after the end of the stimulation. In particular, phase locking of frontoparietal areas with θ-rTMS, the left occipitoparietal area with α-rTMS, and the right motor area with β-rTMS were maintained in both the visual and motor area TMS-target conditions (see magnified topography at post 1.5 cycles in Figures 4A,B).

**Figure 5:**
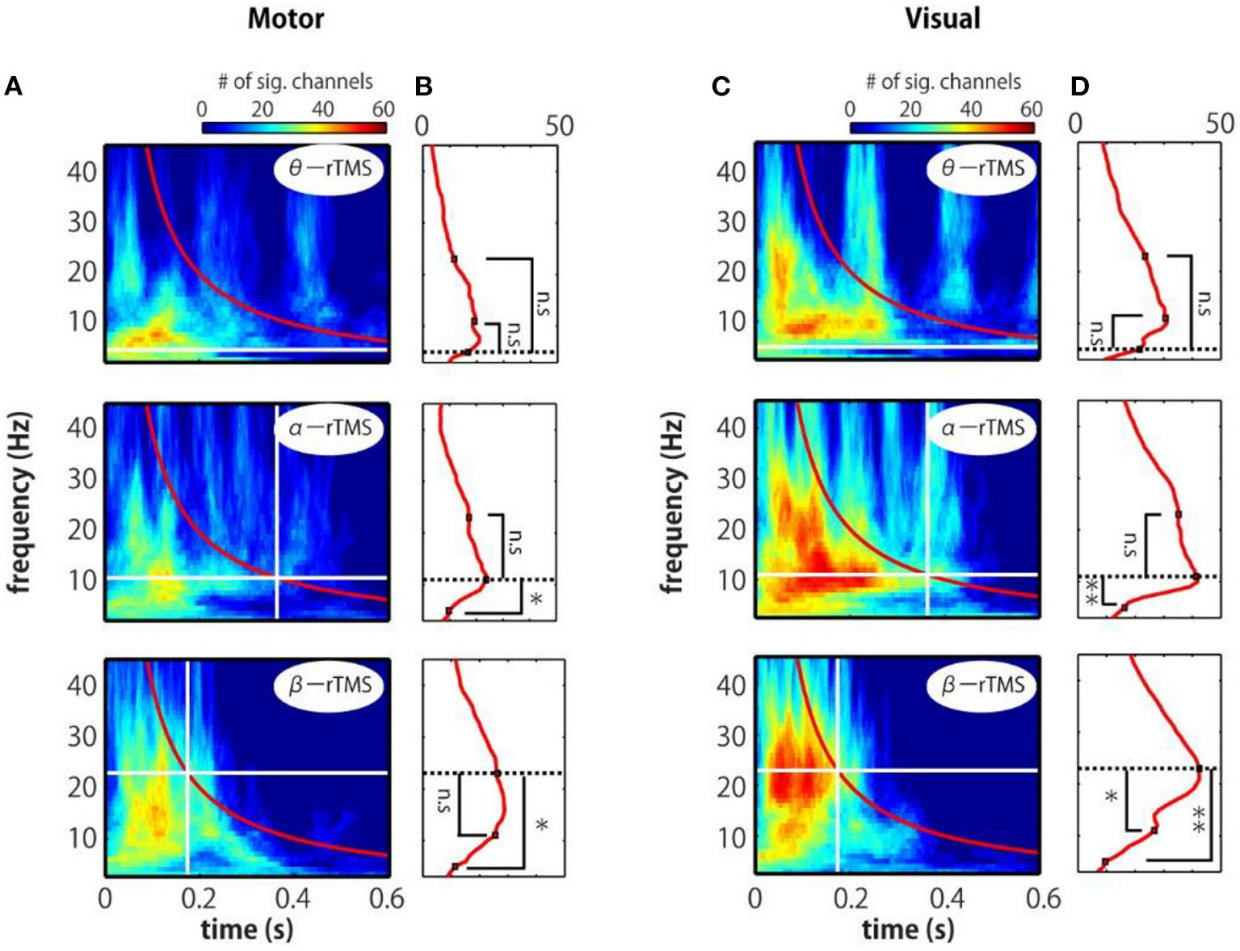
Time-frequency representations of the number of significant electrodes. **(A,C)** Each map indicates the number of significant electrodes, determined by comparing the real and sham conditions. **(B,D)** Mean number of significant electrodes over four cycles at each frequency (red line in panels **A** and **C**). The vertical and horizontal lines indicate the timing of the last pulse and the stimulation frequency. The number of significant electrodes was compared between the stimulation frequency and other frequencies (binomial test; *p < 0.005, **p < 0.001).

We further examined the time–frequency profile of the spatial extent of phase locking from the number of electrodes with significant phase locking. For motor stimulation, phase locking at alpha-band frequencies was induced in many electrodes by α-rTMS (Figures 5A,B, middle panels). On the other hand, β-rTMS induced oscillations at both the alpha- and beta-band frequencies (Figures 5A,B, bottom panels). For α-rTMS or β-rTMS over the visual cortex, the induced oscillations were prominent at the stimulation frequency (Figures 5C,D, middle and bottom panels). To quantify this frequency specificity, we averaged the number of significant electrodes over four cycles at each frequency (indicated by the red lines in Figures 5A,C). We used the binomial test to assess whether more electrodes were phase locked at the stimulation frequency than at the other frequencies; for example, for θ-rTMS, we assessed whether there were significantly more phase-locked electrodes at 5 Hz (the theta band) than at 11 or 23 Hz (see Figures 5B,D). The mean number of electrodes with significant phase locking was greater around the stimulation frequency than at the other frequencies. Namely, for α-rTMS to the motor cortex (Figure 5B, middle panel) and visual cortex (Figure 5D, middle panel), alpha-band neural oscillations were more globally entrained than oscillations in other frequency bands. Similarly, for β-rTMS to the motor cortex (Figure 5B, bottom panel) and visual cortex (Figure 5D, bottom panel), beta-band oscillations were more globally entrained than those in other frequency bands. We also noted that there were significantly more electrodes with significant phase locking for visual cortex stimulation than for motor cortex stimulation (θ-rTMS: *p* = 0.1727; α-rTMS: *p* = 0.0008; β-rTMS: *p* = 0.0019; binomial test). The fact that phase locking beyond the stimulation site was most prominent around the stimulation frequency suggests that globally coupled frequency-specific neural oscillators in brain networks were phase-entrained by rTMS. While sp-TMS also resulted in global phase locking, this was non-frequency-specific (Supplementary Figure 3).

### Elimination of Common Components Induced by Stimulation

**Figure 6:**
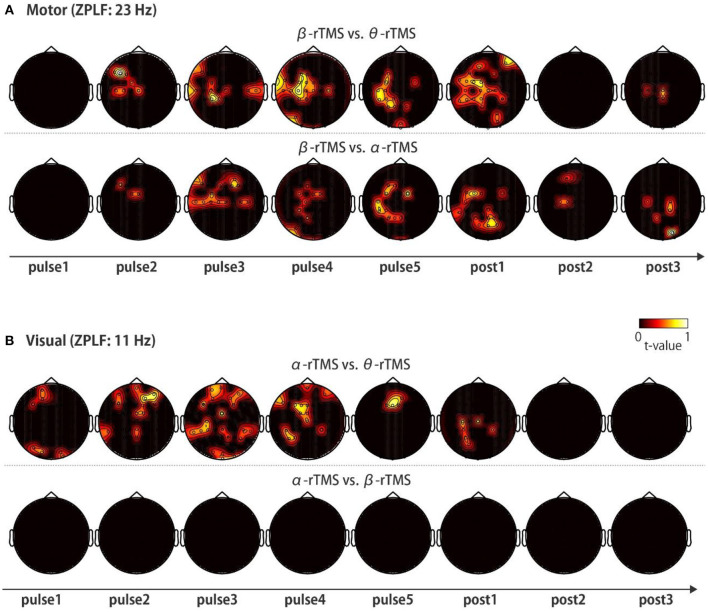
Topographic representation of the ZPLF. Each map with *t*-values was obtained from comparisons of the ZPLF between β-rTMS and the other stimulation frequency, i.e., θ-rTMS (upper panels) and α-rTMS (lower panels) for motor cortex stimulation **(A)**, and between α-rTMS and the other frequency, i.e., θ-rTMS (upper panels) and β-rTMS (lower panels) for visual cortex stimulation **(B)** (*p* < 0.05).

Finally, to investigate the frequency-specific spatial distribution of phase locking, we determined the regions in which non-common phase locking occurred under distinct stimulation conditions. Specifically, we compared the topographies of the ZPLF for each stimulation pulse across θ-rTMS, α-rTMS, and β-rTMS conditions using the cluster-based permutation test. Under motor cortical stimulation conditions (Figure 6A), the β-band ZPLF in β-rTMS was significantly stronger than the beta-band ZPLF in θ-rTMS (Figure 6A, upper panel), and α-rTMS (Figure 6A, lower panel). Interestingly, there was no significant difference in the ZPLF for the first pulse; however, a stronger ZPLF was observed outside the stimulation area after the second pulse and even after stimulation terminated. Under visual cortical stimulation conditions (Figure 6B), α-rTMS resulted in a significantly larger α-band ZPLF than θ-rTMS in widely distributed areas (Figure 6B, upper panel), while it was negligible under the β-rTMS condition (Figure 6B, lower panel). Direct comparisons between rTMS frequency conditions not only revealed frequency-specific responses but also removed non-specific noise and evoked components, which were common across all stimulation conditions. Thus, these topographical representations indicate that rhythmic stimulation induces the spatial spread of frequency-specific phase locking.

## Discussion

### Efficient Stimulation Frequencies for the Phase Entrainment of Intrinsic Oscillations

It has been reported that rhythmic stimulation at a frequency that matches physiological rhythms is the most efficient in modulating behaviors *via* the entrainment of task-related oscillations (Klimesch et al., 2003; Sauseng et al., 2009; Romei et al., 2011). In particular, Klimesch et al. demonstrated that rTMS at the stimulation frequency of IAF +1 Hz was beneficial for cognitive performance, whereas it was ineffective at the stimulation frequency of IAF +3 Hz (Klimesch et al., 2003). More recently, an rTMS-EEG study by Thut et al. showed that intrinsic alpha oscillations were entrained by rTMS at the IAF applied to the alpha source (Thut et al., 2011b). From the perspective of non-linear dynamical systems theory, the degree of entrainment of an oscillatory system to the rhythmic stimulation changes as a function of stimulation frequency and amplitude; this is known as the “synchronization region,” or the “Arnold tongue” (Pikovsky et al., 2003). If the stimulation amplitude is low, only rhythms close to the natural frequency can entrain the system, but as the stimulation amplitude increases, the system is entrained to a wider range of stimulation frequencies. In a model evaluating the degree of entrainment by transcranial alternating current stimulation (tACS) under a comprehensive array of stimulation conditions (amplitude: 1–13 pA; frequency: 0–6 Hz;), tACS matched to the natural frequency was the most efficient in entraining network activity at the lowest amplitude (Ali et al., 2013).

To our knowledge, the present study is the first to demonstrate frequency-specific phase entrainment of ongoing oscillations that correspond to the natural frequency characteristic of each local area; phase entrainment was observed at specific frequencies depending on the stimulation site. Specifically, local activity during α-rTMS over the visual cortex and β-rTMS over the motor cortex exhibited some signatures of phase entrainment, such as a continuous and gradual increase in phase locking and its persistence after the stimulation had ended. The reason why responses to periodic stimulation differed between regions may be because the natural frequency of each region is different, and only stimulation within the synchronization region was effective for entrainment.

### Local Entrainment Signatures

Phase entrainment is established by a sequential phase shift (Lakatos et al., 2019) when there is an effective relationship between the rhythm of the oscillating system and the external force, i.e., within the synchronization region. Thus, it is expected that the phase shift induced by a periodic external force of an appropriate intensity (i.e., not too strong and not too weak) and an appropriate frequency (i.e., near-natural frequency) will result in a gradual increase in phase locking across trials (Thut et al., 2011a). In other words, it takes some time for the oscillatory system and the external rhythm to synchronize completely, and as the frequency of the two differs, the time for synchronization may be prolonged or synchronization might not even occur. Furthermore, when the external force is terminated, the entrained oscillations slowly revert to their natural frequencies (Lakatos et al., 2019). However, the stronger the entrainment to the external rhythm was, the more the phase locking at that frequency sustained. Given the progressive increase in the ZPLF in the alpha and beta bands and the sustainability of the effect at these frequencies, α-rTMS and β-rTMS likely match the natural frequencies of the visual and motor cortices, respectively. However, phase locking did not last under other TMS conditions, probably because the stimulation rhythm and the natural frequencies of the motor and visual cortices were significantly different. In this case, it is likely that they will hardly synchronize, or if they do, the oscillations of the cortical system will return to their natural frequencies more rapidly.

Such lasting effects were consistently apparent, even in the individual natural frequencies of alpha-band oscillations. Specifically, participants with an IAF closer to (not equal to) the stimulation frequency (i.e., closer to 11 Hz; see Supplementary Figure 2) had longer phase locking after the end of stimulation. Conversely, if the stimulation frequency and intensity are within the appropriate range, i.e., within the synchronization region, it is possible to cause entrainment even if the stimulation frequency and the natural frequency are different.

One might ask why phase locking after sp-TMS lasted longer than that after rTMS; for instance, significant phase locking of motor and visual cortex after sp-TMS lasted for about 150 ms (ca. 3 cycles) or about 200 ms (ca. 2 cycles), peaking at 21 and 8 Hz, respectively (see Figures 2A,E). On the other hand, phase locking after β-rTMS and α-rTMS lasted for about 2.5 cycles and about 1.5 cycles, respectively (see Figures 2D,G). The effect of sp-TMS is obviously not phase entrainment. Under the sp-TMS condition, neural oscillations are phase reset once, but they continue to oscillate at the natural frequency (Rosanova et al., 2009; Herring et al., 2015; Lakatos et al., 2019). On the other hand, under the rTMS condition, the ongoing oscillations synchronized with a cycle that was slightly different from the natural frequency. Thus, the prominent frequencies of sp-TMS are different from those of rTMS, and the phase-entrained oscillations return to the intrinsic oscillations a few cycles after rTMS has been terminated. Additionally, we found that phase locking to the theta stimulation frequency was prominent and significant for every TMS pulse (see Figure 3D). This may be attributed to either phase reset by every pulse, as observed in sp-TMS, or by repeated evoked neural components (discussed in section Limitations), or both. Taken together, our results suggest that different local areas have their own natural frequency and that rTMS tuned to frequencies close to the natural frequency of those areas can most efficiently modulate their oscillatory dynamics.

### Local Entrainment Propagates to Other Areas in a Frequency-Specific Manner

We showed that rhythmic stimulation initially causes phase locking over a wide spatial area and a broad range of frequencies, but that phase locking around the stimulation frequency is predominantly propagated as the pulse train continues. Furthermore, the areas to which phase locking eventually propagated differed for each stimulation frequency. These results suggest that local phase entrainment may lead to global phase entrainment of neural oscillators with the same natural frequency in functionally coupled regions.

Prior MEG/EEG studies have demonstrated that functionally relevant networks are spatially organized through a frequency-specific synchronous oscillating activity (Laufs et al., 2003; Hillebrand et al., 2012; Hipp et al., 2012). Thus, frequency-tuned stimulation to the frequency-specific network node may drive chained phase entrainment of coupled neural oscillators from local to distant brain regions. We anticipated that β-rTMS over the motor cortex would be particularly effective because the functional connectivity between the motor area and other cortical areas is largely achieved *via* beta-band oscillations (Hillebrand et al., 2012). However, β-rTMS over the motor cortex increased ZPLF in the alpha band as well as that in the beta band (Figure 5B, bottom panel). The stimulation frequency used in our study (23 Hz) may have been suboptimal for entraining intrinsic oscillations because the intrinsic Rolandic beta frequency over the sensory-motor strip varies among individuals in a broad range from 14 to 30 Hz. Alternatively, the functional connectivity between the motor cortex and other cortices might be lower than that between the visual cortex and other cortices (Hillebrand et al., 2012). This hypothesis is plausible because the primary motor cortex is the final cortical stage for executing a motor output and hence mainly receives inputs from other brain areas, whereas the primary visual cortex is the first cortical stage to receive visual inputs, and it then sends these signals to functionally connected areas.

### Limitations

The number of survived trials (24.8 ± 2.5 trials) per block was relatively small and might have been insufficient to yield significant differences in PLF across conditions. This limitation was due to the already long total length of experiments (4–5 h) and involved a tradeoff with finishing the experiment within a single day to avoid daily variation. Thus, we acknowledge that the small number of trials may have resulted in a statistical type II error; additional trials or samples for conditions or time ranges that were not statistically significant would have overcome this. Furthermore, there were differences in the number of trials that were caused by the removal of artifact-contaminated trials. However, we assessed an unbiased estimator of PLF (i.e., the ZPLF), which corrects for the differences in the number of trials and gives a robust measure for phase locking using even <30 trials, both theoretically and empirically (see Supplementary Figure 1). As the mean and variance in the PLF were corrected across stimulation conditions (areas and frequencies), the number of trials used in the current study should not have biased the ZPLF results.

TMS-induced EEG potentials are composed of transcranial- and non-transcranial-neural activity, i.e., those derived from auditory and somatosensory cortices, and various artifacts, including those caused by signal processing limitations such as the wavelet time window and interpolation. Findings on the increases in the ZPLF during stimulation may be partially due to contamination with repeated evoked neural components and repeated artifacts. If an evoked component with a constant latency and polarity is superimposed on ongoing oscillations, the intertrial phase variability of the EEG signals will decrease as if the ongoing oscillation is aligned to a specific phase (Sauseng et al., 2007). Such spurious phase locking can partially be produced by repeated TMS-related artifacts, as well as by repeated TMS-evoked neural activity.

While it is challenging to distinguish phenomena caused by neural phase entrainment, recurring evoked activities, or repetition of artifacts produced by TMS, phase entrainment signatures such as cumulative phase locking and sustained effects after the end of stimulation cannot be explained by the linear sum of single evoked components (Thut et al., 2011a). Moreover, each cortical system has a specific frequency that is particularly effective for entraining the system. Stimulation with such area-specific frequencies resulted in significantly stronger phase locking than other periodic stimulations; these results are thus unlikely to be the result of TMS artifacts and evoked activity. Furthermore, the significant differences in topographic maps revealed by comparing the ZPLF between stimulus conditions imply the existence of frequency-specific oscillatory networks that are not common components, such as auditory responses, somatosensory responses, or various artifacts caused by each TMS pulse.

Nevertheless, non-transcranial periodic sensory inputs derived from somatosensory and auditory systems *via* bone conduction can indirectly entrain the phase of ongoing oscillations of the motor and visual cortex, much like steady-state somatosensory/auditory evoked potentials). Auditory components were partially attenuated by earplugs and compensated for by including a sham control. However, compared with the direct cortical effects, phase entrainment caused by indirect interactions is unlikely or marginal in terms of temporal accuracy of periodicity. In other words, neural responses to sensory inputs were not identical every time, as observed in many event-related studies. Further studies are needed to clarify whether phase entrainment occurs remotely *via* non-transcranial stimulation to different modalities from the stimulation area. It is also an open question as to how much the direct impacts of cortical stimulation can be maximized in the presence of noise by optimizing experimental and analytic methods, including the inclusion of realistic sham control (Ruohonen et al., 2000; Rossi et al., 2007; Hoeft et al., 2008; Conde et al., 2019) and reference methods (Belardinelli et al., 2019). Although contamination by TMS-related artifacts remains a possibility, we believe that the neural phase entrainment observed in this study is more likely to have been caused by the phase entrainment of ongoing oscillations rather than by repetitive evoked and/or artifact components.

### Potential Applications

It should be noted that it is impossible to dissociate real and spurious connectivity of two cortical regions through conventional phase synchronization analyses if the two regions are driven by a common source (Kitajo and Okazaki, 2016). However, effective connectivity that is indicative of directional causality between two regions can be probed by local perturbations. We, therefore, propose that rhythmic stimulation can be used to probe causal communication between cortical regions that are fueled by oscillatory activity with a specific frequency.

In addition, the present results suggest that rTMS can be used as an efficient tool to manipulate and measure frequency-specific brain dynamics. This application could provide insights into the causal roles of rhythmic activity in brain networks by looking at the functional outcomes of modulating synchronous neural oscillations that mediate specific cognitive functions.

Several brain disorders show impaired neural oscillations and synchrony within brain networks, including schizophrenia (Lee et al., 2006; Uhlhaas et al., 2006) and stroke (Wu et al., 2011; Kawano et al., 2017, 2020). Therefore, this rTMS-EEG technique has clinical potential for the treatment of impaired oscillations and synchrony in such brain disorders. For instance, it has been reported that resting-state interhemispheric alpha-band and beta-band phase synchrony networks are impaired in stroke patients (Kawano et al., 2017). The degree of impairment of the interhemispheric phase synchrony networks was highly correlated with the functional independence measure (FIM), which assesses activities of daily living (ADL) in stroke patients. We, therefore, speculate that alpha- or beta-band rTMS targeting interhemispheric synchrony networks can be a potential neurorehabilitation treatment for ADL-related functional brain networks in stroke.

## Data Availability Statement

The datasets presented in this article are not readily available because the data that support the findings of this study are available from the corresponding author upon reasonable request. Requests to access the datasets should be directed to kkitajo@nips.ac.jp.

## Ethics Statement

The studies involving human participants were reviewed and approved by the RIKEN Ethics Committee. The patients/participants provided their written informed consent to participate in this study.

## Author Contributions

KK and TH conceived and designed the experiments. YN and YM prepared the experiments. YO, YN, KK, and TH collected the data. YO and YN analyzed the data. YO, KK, and TH wrote the manuscript. All authors reviewed the manuscript.

## Conflict of Interest

The authors declare that the research was conducted in the absence of any commercial or financial relationships that could be construed as a potential conflict of interest.

## References

[B1] Ali M. M. Sellers K. K. Frohlich F. (2013). Transcranial alternating current stimulation modulates large-scale cortical network activity by network resonance. J. Neurosci. 33, 11262–11275. 10.1523/JNEUROSCI.5867-12.2013 23825429 PMC6618612

[B2] Belardinelli P. Biabani M. Blumberger D. M. Bortoletto M. Casarotto S. David O.. (2019). Reproducibility in TMS-EEG studies: a call for data sharing, standard procedures and effective experimental control. Brain Stimul. 12, 787–790. 10.1016/j.brs.2019.01.010 30738777

[B3] Bonnefond M. Jensen O. (2012). Alpha oscillations serve to protect working memory maintenance against anticipated distracters. Curr. Biol. 22, 1969–1974. 10.1016/j.cub.2012.08.029 23041197

[B4] Brainard D. H. (1997). The psychophysics toolbox. Spat. Vis. 10, 433–436. 10.1163/156856897X00357 9176952

[B5] Conde V. Tomasevic L. Akopian I. Stanek K. Saturnino G. B. Thielscher A.. (2019). The non-transcranial TMS-evoked potential is an inherent source of ambiguity in TMS-EEG studies. Neuroimage 185, 300–312. 10.1016/j.neuroimage.2018.10.052 30347282

[B6] Deco G. Corbetta M. (2011). The dynamical balance of the brain at rest. Neuroscientist 17, 107–123. 10.1177/1073858409354384 21196530 PMC4139497

[B7] Delorme A. Makeig S. (2004). EEGLAB: an open source toolbox for analysis of single-trial EEG dynamics including independent component analysis. J. Neurosci. Methods 134, 9–21. 10.1016/j.jneumeth.2003.10.009 15102499

[B8] Doesburg S. M. Roggeveen A. B. Kitajo K. Ward L. M. (2008). Large-scale gamma-band phase synchronization and selective attention. Cereb. Cortex 18, 386–396. 10.1093/cercor/bhm073 17556771

[B9] Fisher N. I. (1993). Statistical Analysis of Circular Data. New York, NY: Cambridge University Press. 10.1017/CBO9780511564345

[B10] Fries P. (2005). A mechanism for cognitive dynamics: neuronal communication through neuronal coherence. Trends Cogn. Sci. 9, 474–480. 10.1016/j.tics.2005.08.011 16150631

[B11] Glim S. Okazaki Y. O. Nakagawa Y. Mizuno Y. Hanakawa T. Kitajo K. (2019). Phase-amplitude coupling of neural oscillations can be effectively probed with concurrent TMS-EEG. Neural Plast. 2019:6263907. 10.1155/2019/6263907 31049054 PMC6462323

[B12] Herring J. D. Thut G. Jensen O. Bergmann T. O. (2015). Attention modulates TMS-locked alpha oscillations in the visual cortex. J. Neurosci. 35, 14435–14447. 10.1523/JNEUROSCI.1833-15.2015 26511236 PMC4623224

[B13] Hillebrand A. Barnes G. R. Bosboom J. L. Berendse H. W. Stam C. J. (2012). Frequency-dependent functional connectivity within resting-state networks: an atlas-based MEG beamformer solution. Neuroimage 59, 3909–3921. 10.1016/j.neuroimage.2011.11.005 22122866 PMC3382730

[B14] Hipp J. F. Hawellek D. J. Corbetta M. Siegel M. Engel A. K. (2012). Large-scale cortical correlation structure of spontaneous oscillatory activity. Nat. Neurosci. 15, 884–890. 10.1038/nn.3101 22561454 PMC3861400

[B15] Hoeft F. Wu D. A. Hernandez A. Glover G. H. Shimojo S. (2008). Electronically switchable sham transcranial magnetic stimulation (TMS) system. PLoS ONE 3:e1923. 10.1371/journal.pone.0001923 18398456 PMC2271126

[B16] Kammer T. Beck S. Thielscher A. Laubis-Herrmann U. Topka H. (2001). Motor thresholds in humans: a transcranial magnetic stimulation study comparing different pulse waveforms, current directions and stimulator types. Clin. Neurophysiol. 112, 250–258. 10.1016/S1388-2457(00)00513-7 11165526

[B17] Kawano T. Hattori N. Uno Y. Hatakenaka M. Yagura H. Fujimoto H.. (2020). Electroencephalographic phase synchrony index as a biomarker of poststroke motor impairment and recovery. Neurorehabil. Neural Repair 34, 711–722. 10.1177/1545968320935820 32691673 PMC7457459

[B18] Kawano T. Hattori N. Uno Y. Kitajo K. Hatakenaka M. Yagura H.. (2017). Large-scale phase synchrony reflects clinical status after stroke: an EEG study. Neurorehabil. Neural Repair 31, 561–570. 10.1177/1545968317697031 28506148

[B19] Kawasaki M. Kitajo K. Yamaguchi Y. (2010). Dynamic links between theta executive functions and alpha storage buffers in auditory and visual working memory. Eur. J. Neurosci. 31, 1683–1689. 10.1111/j.1460-9568.2010.07217.x 20525081 PMC2878597

[B20] Kawasaki M. Uno Y. Mori J. Kobata K. Kitajo K. (2014). Transcranial magnetic stimulation-induced global propagation of transient phase resetting associated with directional information flow. Front. Hum. Neurosci. 8:173. 10.3389/fnhum.2014.00173 24723875 PMC3971180

[B21] Kayser J. Tenke C. E. (2006). Principal components analysis of Laplacian waveforms as a generic method for identifying ERP generator patterns: II. Adequacy of low-density estimates. Clin. Neurophysiol. 117, 369–380. 10.1016/j.clinph.2005.08.033 16356768

[B22] Kitajo K. Okazaki Y. O. (2016). TMS-EEG for probing distinct modes of neural dynamics in the human brain. Adv. Cogn. Neurodyn. 5, 211–216. 10.1007/978-981-10-0207-6_30

[B23] Kleiner M. Brainard D. Pelli D. Ingling A. Murray R. Broussard C. (2007). What's new in psychtoolbox-3. Perception 36, 1–16. 10.1177/03010066070360S101

[B24] Klimesch W. Sauseng P. Gerloff C. (2003). Enhancing cognitive performance with repetitive transcranial magnetic stimulation at human individual alpha frequency. Eur. J. Neurosci. 17, 1129–1133. 10.1046/j.1460-9568.2003.02517.x 12653991

[B25] Klimesch W. Schack B. Schabus M. Doppelmayr M. Gruber W. Sauseng P. (2004). Phase-locked alpha and theta oscillations generate the P1-N1 complex and are related to memory performance. Brain Res. Cogn. Brain Res. 19, 302–316. 10.1016/j.cogbrainres.2003.11.016 15062867

[B26] Komssi S. Kahkonen S. Ilmoniemi R. J. (2004). The effect of stimulus intensity on brain responses evoked by transcranial magnetic stimulation. Hum. Brain Mapp. 21, 154–164. 10.1002/hbm.10159 14755835 PMC6871924

[B27] Korhonen R. J. Hernandez-Pavon J. C. Metsomaa J. Maki H. Ilmoniemi R. J. Sarvas J. (2011). Removal of large muscle artifacts from transcranial magnetic stimulation-evoked EEG by independent component analysis. Med. Biol. Eng. Comput. 49, 397–407. 10.1007/s11517-011-0748-9 21331656

[B28] Lachaux J. P. Rodriguez E. Van Quyen M. L. Lutz A. Martinerie J. Varela F. J. (2000). Studying single-trials of phase synchronous activity in the brain. Int. J. Bifurcat. Chaos 10, 2429–2439. 10.1142/S0218127400001560

[B29] Lakatos P. Gross J. Thut G. (2019). A new unifying account of the roles of neuronal entrainment. Curr. Biol. 29, R890–R905. 10.1016/j.cub.2019.07.075 31550478 PMC6769420

[B30] Laufs H. Krakow K. Sterzer P. Eger E. Beyerle A. Salek-Haddadi A.. (2003). Electroencephalographic signatures of attentional and cognitive default modes in spontaneous brain activity fluctuations at rest. Proc. Natl. Acad. Sci. U. S. A. 100, 11053–11058. 10.1073/pnas.1831638100 12958209 PMC196925

[B31] Lee S. H. Wynn J. K. Green M. F. Kim H. Lee K. J. Nam M.. (2006). Quantitative EEG and low resolution electromagnetic tomography (LORETA) imaging of patients with persistent auditory hallucinations. Schizophr. Res. 83, 111–119. 10.1016/j.schres.2005.11.025 16524699

[B32] Litvak V. Komssi S. Scherg M. Hoechstetter K. Classen J. Zaaroor M.. (2007). Artifact correction and source analysis of early electroencephalographic responses evoked by transcranial magnetic stimulation over primary motor cortex. Neuroimage 37, 56–70. 10.1016/j.neuroimage.2007.05.015 17574872

[B33] Luczak A. Bartho P. Harris K. D. (2009). Spontaneous events outline the realm of possible sensory responses in neocortical populations. Neuron 62, 413–425. 10.1016/j.neuron.2009.03.014 19447096 PMC2696272

[B34] Maris E. Oostenveld R. (2007). Nonparametric statistical testing of EEG- and MEG-data. J. Neurosci. Methods 164, 177–190. 10.1016/j.jneumeth.2007.03.024 17517438

[B35] Mazaheri A. Jensen O. (2006). Posterior alpha activity is not phase-reset by visual stimuli. Proc. Natl. Acad. Sci. U. S. A. 103, 2948–2952. 10.1073/pnas.0505785103 16473952 PMC1413767

[B36] Okazaki Y. O. Mizuno Y. Kitajo K. (2020). Probing dynamical cortical gating of attention with concurrent TMS-EEG. Sci. Rep. 10:4959. 10.1038/s41598-020-61590-2 32188883 PMC7080792

[B37] Oostenveld R. Fries P. Maris E. Schoffelen J. M. (2011). FieldTrip: open source software for advanced analysis of MEG, EEG, and invasive electrophysiological data. Comput. Intell. Neurosci. 2011:156869. 10.1155/2011/156869 21253357 PMC3021840

[B38] Pelli D. G. (1997). The VideoToolbox software for visual psychophysics: transforming numbers into movies. Spat. Vis. 10, 437–442. 10.1163/156856897X00366 9176953

[B39] Perrin F. Pernier J. Bertrand O. Echallier J. F. (1989). Spherical splines for scalp potential and current density mapping. Electroencephalogr. Clin. Neurophysiol. 72, 184–187. 10.1016/0013-4694(89)90180-6 2464490

[B40] Pikovsky A. Rosenblum M. Kurths J. (2003). Synchronization - A Universal Concept in Nonlinear Sciences. Cambidge: Cambidge University Press.

[B41] Rodriguez E. George N. Lachaux J. P. Martinerie J. Renault B. Varela F. J. (1999). Perception's shadow: long-distance synchronization of human brain activity. Nature 397, 430–433. 10.1038/17120 9989408

[B42] Romei V. Driver J. Schyns P. G. Thut G. (2011). Rhythmic TMS over parietal cortex links distinct brain frequencies to global versus local visual processing. Curr. Biol. 21, 334–337. 10.1016/j.cub.2011.01.035 21315592 PMC3063337

[B43] Rosanova M. Casali A. Bellina V. Resta F. Mariotti M. Massimini M. (2009). Natural frequencies of human corticothalamic circuits. J. Neurosci. 29, 7679–7685. 10.1523/JNEUROSCI.0445-09.2009 19535579 PMC6665626

[B44] Rossi S. Ferro M. Cincotta M. Ulivelli M. Bartalini S. Miniussi C.. (2007). A real electro-magnetic placebo (REMP) device for sham transcranial magnetic stimulation (TMS). Clin. Neurophysiol. 118, 709–716. 10.1016/j.clinph.2006.11.005 17188568

[B45] Ruohonen J. Ollikainen M. Nikouline V. Virtanen J. Ilmoniemi R. J. (2000). Coil design for real and sham transcranial magnetic stimulation. IEEE Trans. Biomed. Eng. 47, 145–148. 10.1109/10.821731 10721620

[B46] Sauseng P. Klimesch W. Gruber W. R. Hanslmayr S. Freunberger R. Doppelmayr M. (2007). Are event-related potential components generated by phase resetting of brain oscillations? A critical discussion. Neuroscience 146, 1435–1444. 10.1016/j.neuroscience.2007.03.014 17459593

[B47] Sauseng P. Klimesch W. Heise K. F. Gruber W. R. Holz E. Karim A. A.. (2009). Brain oscillatory substrates of visual short-term memory capacity. Curr. Biol. 19, 1846–1852. 10.1016/j.cub.2009.08.062 19913428

[B48] Siegel M. Donner T. H. Engel A. K. (2012). Spectral fingerprints of large-scale neuronal interactions. Nat. Rev. Neurosci. 13, 121–134. 10.1038/nrn3137 22233726

[B49] Smith S. M. Fox P. T. Miller K. L. Glahn D. C. Fox P. M. Mackay C. E.. (2009). Correspondence of the brain's functional architecture during activation and rest. Proc. Natl. Acad. Sci. U. S. A. 106, 13040–13045. 10.1073/pnas.0905267106 19620724 PMC2722273

[B50] Tallon-Baudry C. Bertrand O. Delpuech C. Pernier J. (1996). Stimulus specificity of phase-locked and non-phase-locked 40 Hz visual responses in human. J. Neurosci. 16, 4240–4249. 10.1523/JNEUROSCI.16-13-04240.1996 8753885 PMC6579008

[B51] Thut G. Schyns P. G. Gross J. (2011a). Entrainment of perceptually relevant brain oscillations by non-invasive rhythmic stimulation of the human brain. Front. Psychol. 2:170. 10.3389/fpsyg.2011.00170 21811485 PMC3142861

[B52] Thut G. Veniero D. Romei V. Miniussi C. Schyns P. Gross J. (2011b). Rhythmic TMS causes local entrainment of natural oscillatory signatures. Curr. Biol. 21, 1176–1185. 10.1016/j.cub.2011.05.049 21723129 PMC3176892

[B53] Uhlhaas P. J. Linden D. E. Singer W. Haenschel C. Lindner M. Maurer K.. (2006). Dysfunctional long-range coordination of neural activity during Gestalt perception in schizophrenia. J. Neurosci. 26, 8168–8175. 10.1523/JNEUROSCI.2002-06.2006 16885230 PMC6673788

[B54] Varela F. Lachaux J. P. Rodriguez E. Martinerie J. (2001). The brainweb: phase synchronization and large-scale integration. Nat. Rev. Neurosci. 2, 229–239. 10.1038/35067550 11283746

[B55] Wu W. Sun J. Jin Z. Guo X. Qiu Y. Zhu Y.. (2011). Impaired neuronal synchrony after focal ischemic stroke in elderly patients. Clin. Neurophysiol. 122, 21–26. 10.1016/j.clinph.2010.06.003 20591730

