# Supplementary material for: Frequency- and Area-Specific Phase Entrainment of Intrinsic Cortical Oscillations by Repetitive Transcranial Magnetic Stimulation
Source: Front Hum Neurosci. 2021 Mar 12;15:608947. doi: 10.3389/fnhum.2021.608947 (PMC7994763; doi:10.3389/fnhum.2021.608947)
Supplement: Supplementary file 1 [file Data_Sheet_1.DOCX]

**Supplementary methods and figures**

## *Sample size bias in PLF and ZPLF*

We tested if ZPLF, which should be an unbiased measure of PLF, is bias-free in terms of the mean and variance even within the range of the number of trials (24.8 ± 2.5: mean ± SD) in the current study using empirical data. To confirm the bias in PLF caused by the sample size, the PLF for each of 1 to 100 sample sizes (the number of trials) for EEG signals from electrode Cz were calculated. The data samples were randomly selected from the pool of pre-TMS time data periods (-1.5 - -0.5) for all stimulation conditions (rTMS, sTMS, Sham). Then, ZPLF was obtained as N x PLF^2, where N is the sample size. Supplementary figure 1 shows the mean (blue line) and 95 % confidence interval (blue area) of the PLF and ZPLF obtained by repeating this procedure 1000 times. The critical value at α = 0.05 (significance level) for PLF and ZPLF can be obtained by $\sqrt{{-ln p}/N}$ and $-ln p$, respectively, where p = 0.05 and N is the number of trials used to calculate PLF and ZPLF ([Fisher, 1993](#_ENREF_1)). Since the mean and variance of ZPLF were steady as a function of the sample size, including the range of the current study (24.8 ± 2.5: mean ± SD), we conclude that the number of trials did not bias the ZPLF results in the current study.

## *Individual alpha frequency (IAF)*

The entrainment effect was also examined at individual natural frequencies, i.e. individual alpha frequencies. The FFT-based power spectrum was computed from pre-stimulation data points [-2 - 0] of sham conditions (Hanning window, frequency range: 2–45 Hz, electrode: Oz). A peak in the power spectrum was identified between 6–13 Hz and defined as the IAF. While most participants showed a single clear peak, four participants showed multiple obscure peaks. The latter were excluded from the this IAF analysis. Participants were divided into two low-IAF and high-IAF groups according to ascending IAF as shown in supplementary figure 2A. The difference between IAF and stimulation frequency was smaller in the high-IAF group than in the low-IAF group. Supplementary figure 2B shows a ZPLF with 11 Hz oscillations at electrode Oz for each group. Under the α-rTMS condition, ZPLF during stimulation did not differ between groups, but ZPLF after stimulation was marginally higher in the high IAF group (*p* < 0.05, uncorrected *t*-test). In other words, the increase in the ZPLF by α-rTMS lasted longer in the group whose IAF was close to the stimulation frequency.


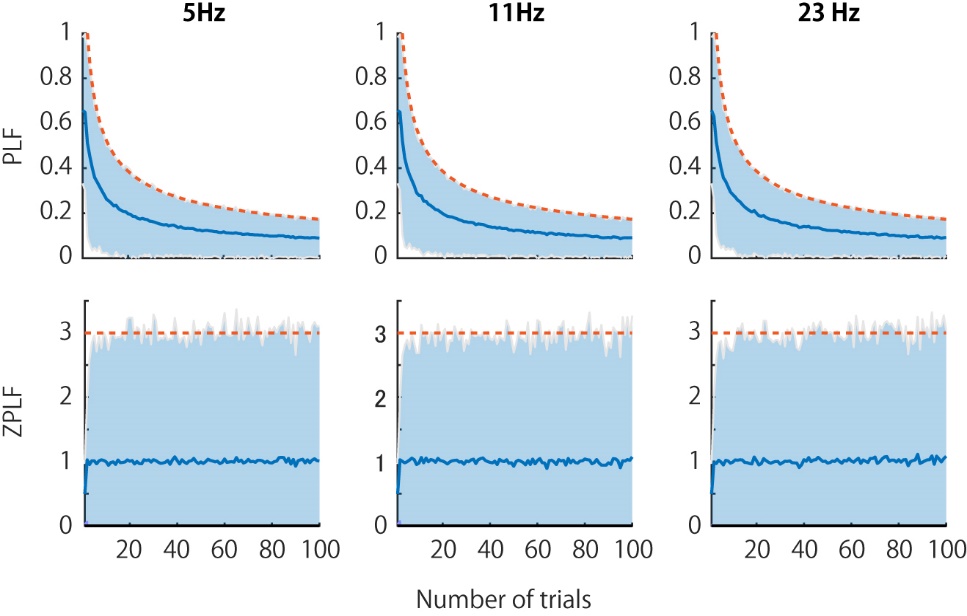


Supplementary figure 1. PLF (A) and ZPLF(B) as a function of the number of trials for a single subject. PLF and ZPLF at the pre-TMS time point (electrode: Cz) were computed with randomly selected trials from a trial pool of all conditions and were averaged over 1000 times iterations. The blue line and blue area are the mean and 95% confidence intervals, respectively. The red line shows the critical PLF and ZPLF values obtained by $\sqrt{{-ln p}/n}$ and $-ln p$, respectively, where *p* = 0.05 and *n* is the number of trials used to calculate PLF and ZPLF.


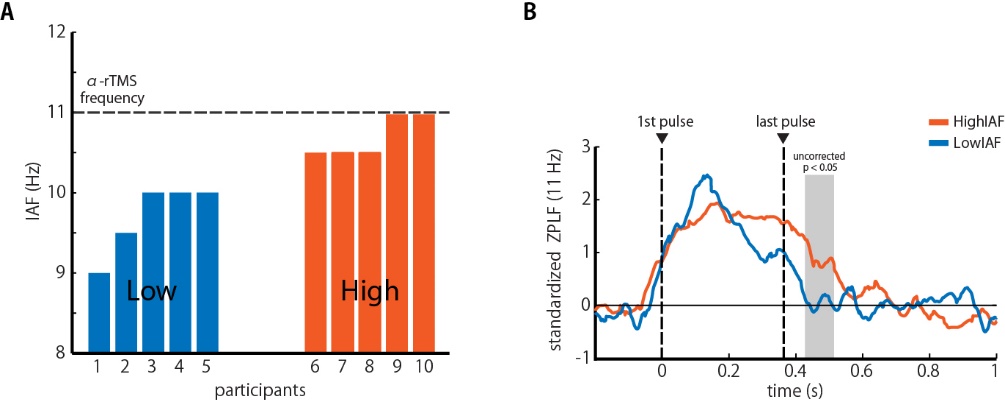


Supplementary figure 2. ZPLF of high-IAF and low-IAF groups. (A) Two groups were separated based on IAF from the Oz channel. Accordingly, the stimulation frequency in the α-rTMS condition was close to the natural frequency of the high-IAF group. (B) The group averaged standardized ZPLF for each group was significantly different in the shaded time range (*p* < 0.05, uncorrected *t*-test).

*
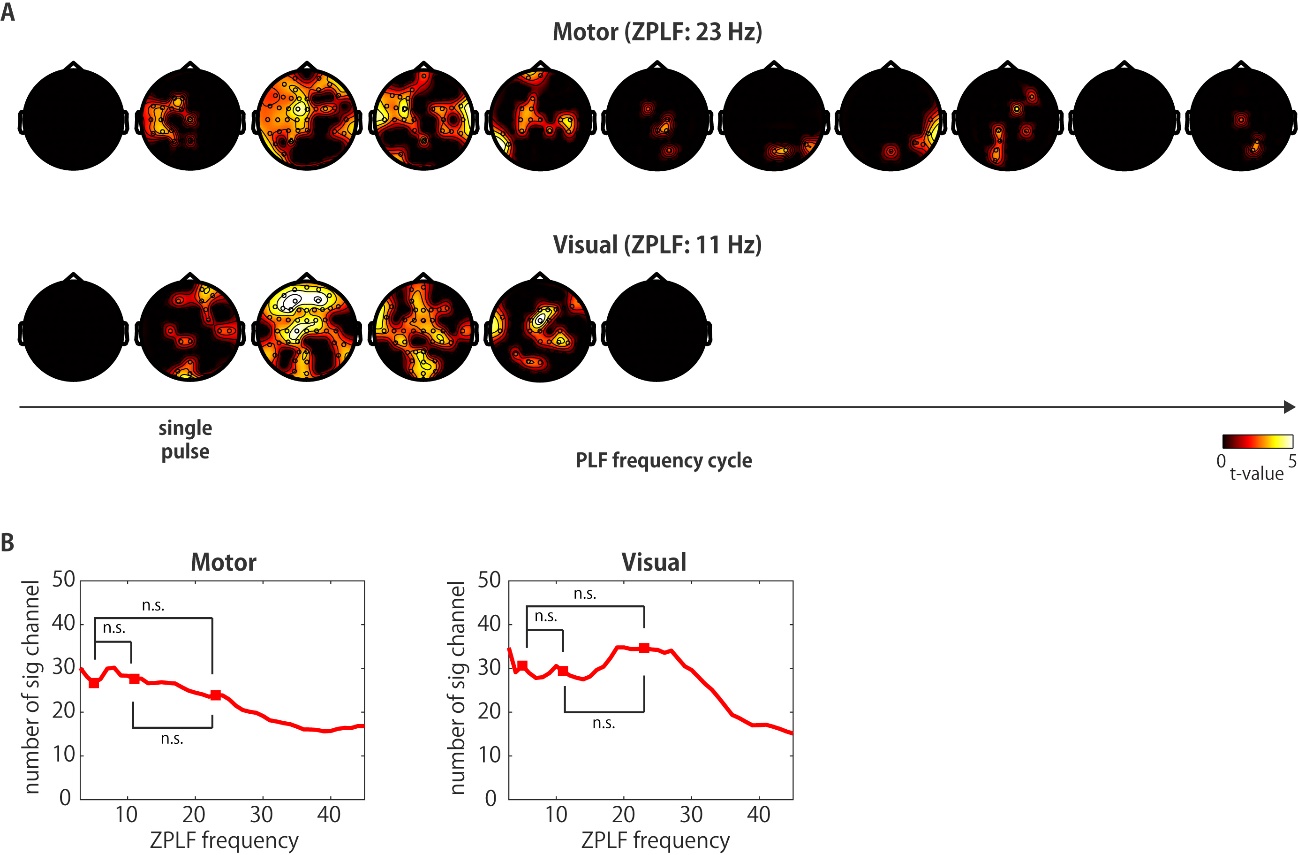
*

Supplementary figure 3. Global phase locking by sTMS. (A) Each topographical map with *t*-values indicates significantly larger ZPLF for motor (upper maps) and for visual (lower maps) stimulation than under the sham condition (*p* < 0.05, cluster-based permutation test). Phase locking of beta oscillations induced in the motor cortex reached the contralateral motor area in at least two cycles and affected a large area over a long period of time. Phase locking of alpha oscillations induced in the visual cortex immediately affected large areas, most prominently the frontal areas. (B) The mean number of significant channels over four cycles at each frequency. The number of significant channels were compared between 5 Hz, 11 Hz, and 23 Hz (n.s., binomial test). In contrast to rTMS, the propagation of phase locking by sTMS was non-frequency-specific.

References

Fisher, N.I., 1993. Statistical analysis of circular data. Cambridge University Press, New York.
